# Post-crizotinib management of effective ceritinib therapy in a patient with *ALK*-positive non-small cell lung cancer

**DOI:** 10.1186/s12885-016-2636-z

**Published:** 2016-08-02

**Authors:** Brian Won, Isa Mambetsariev, Ravi Salgia

**Affiliations:** 1 Section of Hematology/Oncology, University of Chicago, 5841 South Maryland Avenue, MC 2115, Chicago, IL 60637 USA; 2 Current address, Department of Medical Oncology and Therapeutics Research, City of Hope, 1500 E Duarte Rd, Duarte, CA 91010-3000 USA

**Keywords:** *ALK*-positive NSCLC, Ceritinib, Gastrointestinal tolerance, Dose, Case report

## Abstract

**Background:**

We report the re-biopsied diagnosis of a patient with anaplastic lymphoma receptor tyrosine kinase (*ALK*)-positive lung adenocarcinoma successfully treated with ceritinib 450 mg/day taken with food following disease progression and gastrointestinal intolerance to crizotinib.

**Case presentation:**

A 74-year old female patient initially diagnosed with *ALK*-negative lung adenocarcinoma responded to initial standard chemotherapy. The patient was subsequently re-tested by next generation sequencing (NGS) and found to have *ALK* EIF2AK3-ALK fusion, and responded to crizotinib, but ultimately progressed and showed intolerance to this ALK inhibitor. She was then successfully treated with ceritinib 450 mg/day taken with food, has not suffered from any further gastrointestinal side-effects, and remains on ceritinib treatment after 12 months.

**Conclusions:**

Second-line ceritinib treatment, when administered at 450 mg/day with food, is both well tolerated and efficacious in a patient with previously treated lung adenocarcinoma who had discontinued crizotinib due to disease progression and gastrointestinal adverse effects (AEs).

## Background

The treatment of non-small cell lung cancer (NSCLC) has now changed dramatically in recent years and is no longer an unspecific approach based on platinum doublet chemotherapy when patients are molecularly tested and diagnosed with actionable mutations. For example, anaplastic lymphoma receptor tyrosine kinase (*ALK*) gene rearrangement occurs in around 5 % of NSCLC cases [1], and is detected by several methods, including fluorescence in situ hybridization (FISH), immunohistochemistry (IHC), quantitative polymerase chain reaction (qPCR), next generation sequencing (NGS), and reverse transcription polymerase chain reaction of cDNA (RT-PCR) [2]. Out of those methods, FISH is the gold standard in detecting *ALK* rearrangement in NSCLC due to its detection of rearrangements regardless of what the variant and fusion partner, use of archival formalin-fixed paraffin embedded tissue, low tumor cell requirement (~100 tumor cells), clinical validation, and its FDA approval for clinical applications [2]. However, FISH is costly, does not identify specific rearrangements, may miss rare rearrangements, and requires specialized training [2]. Next-generation sequencing (NGS) is another method in detecting *ALK* rearrangements and refers to a variety of platforms that parallel sequences multiple analytes simultaneously; NGS has a relatively quick turnaround, low cost, and high coverage [3]. In one case, Ali et al. identified 31 *ALK* positive patients who had both NGS and FISH testing done [4]. Out of those 31 patients, 11 (35 %) were previously *ALK* FISH negative, indicating that NGS holds value in *ALK* testing in comparison with FISH [4]. Crizotinib (Xalkori®) is approved as first-line treatment when *ALK*-rearrangement as detected by FISH [5]. However, despite initial response rates of 60 % and a median PFS of 8–10 months [1, 6], patients tend to relapse after 1–2 years, often limiting its long-term use [7, 8]. This relapse is often due to developed resistance to crizotinib where in approximately 30 % of resistance cases, patients’ tumors have developed secondary mutations within the kinase domain of ALK [7, 9, 10]. Common resistance mutations include gatekeeper L1196M, G1269A, and G1202R mutations [11]. Other mechanisms of crizotinib resistance are due to amplification of the ALK fusion gene, which can occur alone or in combination with secondary resistance mutations [8, 9]. Last, mechanisms of resistance have been attributed to alternative or bypass signaling pathways, such as the epidermal growth factor receptor (EGFR) and insulin-like growth factor-1 (IGF1R) pathways [7, 10]. Ceritinib (Zykadia^TM^) is a once daily oral, small-molecule, adenosine triphosphate (ATP)-competitive ALK inhibitor that is 20 times more potent against ALK than crizotinib in preclinical studies, with activity against common secondary *ALK* mutations that confer resistance to crizotinib [12]. Ceritinib was granted accelerated approval by the US FDA in 2014 for treating metastatic, *ALK*-positive NSCLC that has progressed on or is intolerant to crizotinib, based on response rate and duration of response [13]. Ceritinib is currently recommended at a dose of 750 mg/day, administered orally on an empty stomach (not within 2 h of a meal) [13]. In a Phase I study in ALK inhibitor-treated and -naïve patients with NSCLC, overall response rates (ORRs) of 56 % and 72 % respectively, were achieved, with a median duration of response of 9.7 months. Median progression free survival (PFS) was 6.9 months in prior ALK inhibitor-treated patients and 18.4 months in ALK inhibitor-naïve patients [14]. In this case report, we present a female patient initially diagnosed with *ALK*-negative lung adenocarcinoma that responded to standard chemotherapy, but was subsequently re-tested by NGS and found to be *ALK*-positive. The patient responded to crizotinib but ultimately progressed and showed gastrointestinal intolerance to the drug. She has been treated successfully with ceritinib 450 mg/day taken with food and remains on ceritinib treatment after 12 months.

## Case presentation

A 74 year-old Caucasian female never-smoker initially presented with a pleural effusion and was diagnosed with invasive adenocarcinoma following a pleural biopsy. The pleural biopsy performed revealed dense fibrous tissue with invasive adenocarcinoma with mucin production and associated psammomatous calcifications. The patient was initially treated with carboplatin, paclitaxel, and bevacizumab with good response, and she continued on maintenance bevacizumab for four years after diagnosis. Subsequently treatment was switched to pemetrexed but had to be stopped due to secondary fatigue and failure to thrive. Molecular (*EGFR*/*ALK*) testing was carried out on tissue procured from the initial right pleural biopsy. Using this sample with 35 % viable tumor tissue, the patient was found to be both *EGFR* wild-type and *ALK*-negative by FISH.

**Fig. 1:**
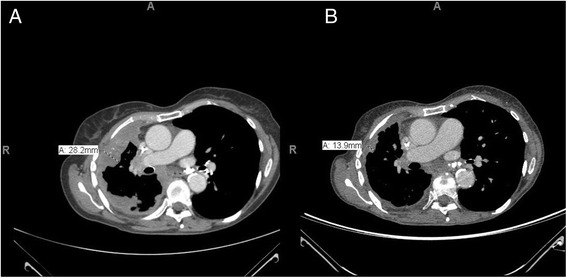
Chest CT Scans of Patient on Crizotinib Treatment. **a** Chest/Upper Abdomen CT Scan Prior to Crizotinib Treatment. **b** Chest CT Scan Post-Crizotinib Treatment

Chest computed tomography (CT) scan showed that the patient had marginal progression of her right pleural rind along with slow progressive growth of right chest wall nodule. She also received palliative radiation to her right chest nodule at this time. In year six, a re-biopsy was carried out on the chest wall skin, and the results indicated the presence of a well differentiated adenocarcinoma, with mucinous features. Immunohistochemistry stains were positive for nuclear thyroid transcription factor-1 and negative for estrogen receptor and progesterone receptor, which were consistent with a lung primary tumor. The tissue obtained was analyzed by NGS to identify potential actionable mutations. NGS testing results (Foundation Medicine, Inc., MA) indicated that the tumor was *ALK*-rearrangement positive for EIF2AK3-ALK fusion, and the patient received crizotinib treatment for 19 months. The patient responded well to crizotinib in the first 12 months of treatment (Fig. 1), with significant interval decrease in the pleural based tumor, mediastinal lymphadenopathy, and subcutaneous metastatic deposits. Continued decreases in the size of nodules associated with the right pleural-based tumor, mediastinal lymphadenopathy and subcutaneous metastatic deposits was seen, followed by stable disease until 16 months into treatment, when CT showed disease progression. This manifested as an increase in right diaphragmatic pleural-based masses, an increase in right lateral chest wall tumor, and an increase in subcarinal lymphadenopathy (from prior study). 18 months after the start of crizotinib treatment, another CT scan showed a significant response (this was the last CT scan while on crizotinib treatment), with significant interval decrease in size of diaphragmatic pleural-based masses and minimal interval decrease in pleural thickening as described above. Despite the patient’s overall pronounced response to crizotinib, the treatment was eventually discontinued due to disease progression and gastrointestinal adverse events of Grade 3 nausea and vomiting.

**Fig. 2:**
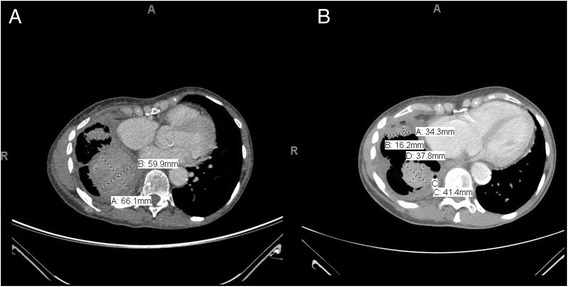
Chest CT Scans of Patient on Ceritinib Tratment. **a** Chest CT Scan Prior to Ceritinib Treatment. **b** Chest/Upper Abdomen CT Scan Post Ceritinib Treatment

Treatment with orally administered ceritinib at a starting dose of 450 mg/day was initiated two months after crizotinib treatment ended. This ceritinib dose was administered with food; meals were generally relatively bland and small in size (taken three times a day), and red meat and spice-free. Ceritinib treatment is being well tolerated, and no gastrointestinal adverse events (nausea, vomiting, or related bowel problems) have been reported to date. Proactive treatment regimens have been reported as effective in the management of gastrointestinal AEs in patients taking ceritinib [15], but regimens of this type were not required for this patient, who was treated with ceritinib 450 mg/day with food, and are not currently considered necessary for her future treatment. Overall, the patient has reported suffering from minimal side effects with the exception of some bloating and rhinorrhea. The patient has also shown a good response to ceritinib, as demonstrated by CT scans (Fig. 2), and treatment is currently ongoing. There was no central nervous system (CNS) involvement.

## Conclusions

**Fig. 3:**
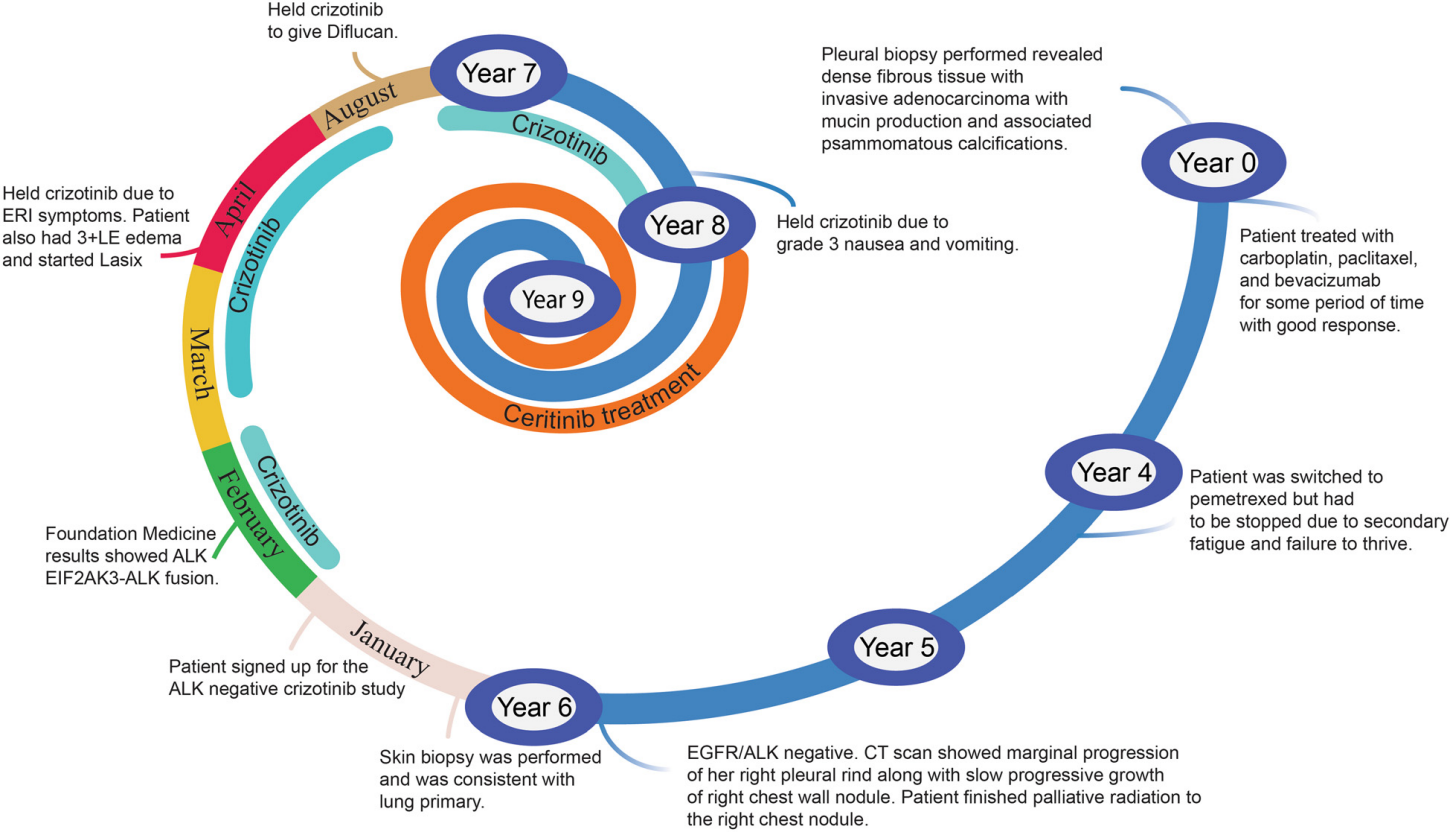
Timeline of Patient’s Oncologic History

In this case study, an excellent response to sequential ALK inhibitor treatment was achieved following re-biopsy in a patient previously shown to be *ALK*-negative. It is not known how common ALK-rearrangements are after treatment with standard chemotherapy or radiotherapy. As data matures from various trials and clinical experience, further tumor heterogeneity will be discerned. It is indeed possible that ALK-rearrangement may be selected for over time. In this patient, NGS testing revealed *ALK-*rearranged adenocarcinoma, which had not been previously detected by FISH. After responding to crizotinib treatment, the patient eventually discontinued the drug due to progressive disease and gastrointestinal adverse events, and therapy was switched to second-line ceritinib. The patient responded to treatment with ceritinib 450 mg/day, taken with food, without suffering from gastrointestinal adverse events that are commonly experienced by patients taking the recommended fasting dose of ceritinib of 750 mg/day [13]. Figure 3 summarizes the patient’s oncologic history as a timeline.

This case study validates the importance of broad diagnostic testing for actionable mutations in lung cancer patients who have progressed on first-line therapy, including in those who may have already been tested and found to be negative for oncogenic driver mutations. For instance, re-biopsy may often provide specimens of superior quality for molecular testing if original tumor biopsy samples were heterogeneous in nature. Mutations not previously detected may also have arisen during treatment. Targeted treatment options are available for those patients where actionable mutations are revealed, and as in this case, crizotinib provides responses in many patients found to be *ALK*-rearrangement positive. However, crizotinib treatment may need to be discontinued due to progression through drug resistance caused by either secondary kinase mutations or alternative genetic driver mutations. As an increasing number of ALK inhibitors are approved for the treatment of patients with lung cancer, such as crizotinib, ceritinib, and alectinib, the selection of sequential treatments will need to be based not only on patient’s resistance mutation profiles but also on consideration of potential tolerability to subsequent treatment options.

This study demonstrates the safety, tolerability and efficacy of second-line ceritinib treatment, when administered with food at a lower than recommended dose of 450 mg/day, in a patient with previously treated lung adenocarcinoma who had discontinued crizotinib due to disease progression and gastrointestinal adverse events. Sequentially administered ceritinib at this lower dose is being well tolerated in this responding patient, with no gastrointestinal intolerance, and she remains on ceritinib therapy to date. As recent data has become available from the J-ALEX study (ASCO, 2016) [16], it may very well be that alectinib will be one of the first line choices for ALK positive patients. As well, there are ongoing studies on Ceritinib in comparison to crizotinib, and these results are awaited. It is an exciting time for our patients with ALK positive tumors with a number of options. How we determine the sequence of therapy is still under investigation. Ceritinib, discussed here, certainly has dramatic activity against ALK positive lung cancer.

## Abbreviations

ALK, anaplastic lymphoma kinase; CNS, central nervous system; CT, Computed tomography; EGFR, epidermal growth factor receptor; FISH, fluorescence in situ hybridization; NGS, next-generation sequencing; NSCLC, non-small cell lung cancer; ORR, overall response rate; PFS, progression-free survival
